# Construction of a prognostic model based on cuproptosis-related genes and exploration of the value of DLAT and DLST in the metastasis for non-small cell lung cancer

**DOI:** 10.1097/MD.0000000000040727

**Published:** 2024-12-06

**Authors:** Huiying Ma, Yizhi Ge, Yuhong Li, Tingting Wang, Wei Chen

**Affiliations:** a Department of Radiation Oncology, The First People’s Hospital of Jiande, Hangzhou, China; b Department of Radiation Oncology, The Affiliated Cancer Hospital of Nanjing Medical University & Jiangsu Cancer Hospital & Jiangsu Institute of Cancer Research, Nanjing, Jiangsu, China.

**Keywords:** cuproptosis, immunotherapy, metastasis, non-small cell lung cancer, prognosis

## Abstract

**Background:**

To reveal the clinical value of cuproptosis-related genes on prognosis and metastasis in non-small cell lung cancer.

**Methods:**

Gene expression profiles and clinical information of non-small cell lung cancer were downloaded from The Cancer Genome Atlas and Gene Expression Omnibus databases. The data were grouped into training set, internal testing set, and external testing set. A risk prognostic model was constructed by Lasso-Cox regression analysis. Hub genes were identified and evaluated using immunohistochemistry and the transwell migration assay in 50 clinical patients.

**Results:**

A total of 17/19 cuproptosis-related genes were differentially expressed in tumors, 8 were significantly associated with prognosis, and 4 were markedly associated with metastasis. A risk model based on 2 cuproptosis-related genes was constructed and validated for predicting overall survival. The risk score was proven to be an independent risk factor for the prognosis of non-small cell lung cancer. Dihydrolipoamide S-acetyltransferase and dihydrolipoamide S-succinyltransferase, key genes in cuproptosis, were proven to be associated with non-small cell lung cancer prognosis and metastasis. Immunohistochemistry showed that their expression significantly predicted metastasis but failed to predict prognosis in non-small cell lung cancer patients. The transwell migration assay further increased the cellular reliability of our findings.

**Conclusion:**

The cuproptosis-related genes prognostic model effectively predicted the prognosis of non-small cell lung cancer. Dihydrolipoamide S-acetyltransferase and dihydrolipoamide S-succinyltransferase may serve as predictive markers for metastasis in non-small cell lung cancer.

## 1. Introduction

Lung cancer remains the deadliest malignancy in the world although much advances in immunotherapy and targeted therapies have been made. There are 28% of lung cancer patients have metastases at the time of diagnosis. Even locally advanced or early stages at diagnosis, they may still develop metastases during the disease progression. This partly cause dismal 5-year survival rate, from 60% of localized lesion to only 6% of metastases in lung cancer.^[1]^ Non-small cell lung cancer (NSCLC) is the most common pathological type, accounting for approximately 80% lung cancer. The prognosis of NSCLC is highly correlated with the appearance of metastasis. However, most metastases in NSCLC are not diagnosed until the metastatic tumor grows to a certain size, making it difficult for clinicians to intervene in the early stages. Early identification of patients with high metastasis risk is the key to improving the survival rate in NSCLC. In the treatment and prognosis of NSCLC, known biomarkers such as epidermal growth factor receptor (EGFR) mutations, Kirsten Rat Sarcoma Viral Oncogene mutations, anaplastic lymphoma kinase rearrangements, and the level of programmed death-ligand 1 (PD-L1) expression play important roles. High PD-L1 expression is closely related to poorer overall survival (OS) rates. These markers are crucial for devising personalized treatment strategies. However, new biomarkers, especially those related to the newly discovered pathway of cuproptosis, may provide a more comprehensive understanding of the disease and additional clinical applications.

Copper (Cu) is an essential micronutrient in physiology, involved in cell proliferation and death pathways. Moreover, Cu has been shown to be strongly associated with tumor progression and metastasis.^[2]^ An imbalance of Cu can induce cell death through targeting lipoylated components of the tricarboxylic acid (TCA) cycle, which is known as cuproptosis.^[3]^ This is a new form of regulated cell death, which is distinguished from known cell death modes such as apoptosis, pyroptosis, necroptosis, autophagy, and ferroptosis. Recent studies have constructed prognostic models based on cuproptosis-related genes (CRGs) and confirmed that these models work well in the prognosis of various tumors.^[4–7]^ However, studies on CRGs in NSCLC are less reported. Dihydrolipoamide S-acetyltransferase (DLAT) and dihydrolipoamide S-succinyltransferase (DLST) are important lipoylated protein genes in the TCA cycle. However, there are no studies have yet to study the clinical value of DLAT and DLST in metastasis of NSCLC.

Therefore, this study was designed to explore the differentially expressed CRGs in NSCLC and metastatic subgroup, and to establish a risk prognostic model. In addition, the predictive value of DLAT and DLST on the prognosis and metastasis of NSCLC was evaluated.

## 2. Materials and methods

### 2.1. Identification of differentially expressed CRGs and prognosis-related CRGs

Gene expression profiles and clinical information of patients with NSCLC were downloaded from The Cancer Genome Atlas (TCGA, https://portal.gdc.cancer.gov/) and gene expression omnibus (GEO, https://www.ncbi.nlm.nih.gov/geo/). The limma package in R software (R version 4.1.3) was applied to normalize the gene expression data. The caret package was applied to randomly divide the NSCLC data from TCGA database into a training set and an internal testing set (1:1 ratio) for subsequent data processing. Perl (version 5.32.1) was used to collate the clinical details. A total of 19 CRGs were identified by literature reviewing. The Wilcoxon Test was used to identify CRGs differentially expressed genes (DEGs) in tumor and normal tissues from TCGA database (*P* < .05, |log2 Fc|≥1), and subsequently to identify CRGs differentially expressed between the M0 and the M1 subgroup similarly. Then survival analysis was performed to identify the prognosis-related CRGs.

### 2.2. Clinical data collection

Fifty NSCLC tissues confirmed by pathology were collected from Jiangsu Cancer Hospital for validation. Twenty-five NSCLC had metastasis and 25 NSCLC had no metastasis, including 45 cases of adenocarcinoma and 5 cases of squamous cell carcinoma. The tissues selection reflects regional disease distribution characteristics but also brings limitations due to uneven subtype distribution. The tissues we collected were sourced from individuals diagnosed before receiving any treatment. This approach helps ensure that the data obtained reflect the natural biological characteristics of the tumors, unaffected by any treatment measures. Immunohistochemical staining was performed on these 50 NSCLC samples, and a complete clinical follow-up of the patients was performed. All procedures were approved by the Ethics Committee of Jiangsu Province Cancer Hospital.

### 2.3. Tumor clustering analysis based on CRGs

The Consensus Cluster Plus package was used to divide the NSCLC from TCGA into different clusters. OS rates were compared between subclusters. Then a heatmap was used to depict the clinicopathological parameters and CRGs expression of different clusters. The gene set variation analysis (GSVA) package was used to performing GSVA for the differential pathways. DEGs between subclusters were identified (|log FC| ≥ 0.585, *P* < .05) and then analyzed for gene ontology and KEGG enrichment.

### 2.4. The construction, validation, and evaluation of the prognostic risk model

The DEGs were firstly examined by univariate Cox regression analysis to obtain prognosis-related DEGs. These prognosis-related DEGs were then analyzed by Lasso Cox regression analysis and cross-validation using glmet package in TCGA training set. Eventually a risk score prognostic model based on CRGs was constructed. Risk scores were calculated for each individual according to the prognostic model formula, and a predictive nomogram of OS was created using clinical variables and risk scores. The patients were classified into high- or low-risk groups based on the median risk score. To evaluate the predictive performance of the model, survival analysis and receiver operating characteristic (ROC) analysis were performed in the TCGA internal testing set and GEO external testing set respectively. The scores of stromal cells and immune cells were obtained based on the ESTIMATE algorithm. The ESTIMATE score was obtained by adding the scores of stromal and immune cells. Finally, the immune checkpoint genes and drug sensitivity were compared between high- and low-risk groups.

### 2.5. Combined diagnosis and single-gene batch correlation analysis for DLAT and DLST

DLAT and DLST are essential lipoylated protein genes in CRGs, and they also play an important role in the prognosis and metastasis of NSCLC. To infer whether they can predict metastasis in NSCLC, a model was constructed using binary logistic regression in Statistical Package for the Social Sciences. To further confirm the reliability of the result, single-gene batch correlation analysis for DLAT and DLST was performed, respectively. Spearman correlation analysis was used to obtain the related genes with DLAT and DLST expression, respectively, and they were listed based on the absolute correlation coefficient. Enrichment analysis was performed on the top 1000 most highly correlated genes.

### 2.6. Immunohistochemistry (IHC) validation

IHC was performed on 50 formalin-fixed, paraffin-embedded NSCLC tumor tissues. The IHC results were analyzed semiquantitatively by using the histochemistry score (H-Score). The IHC technique and the H-Score calculation were as previously reported.^[8]^ The median of the H-Score was used as the cutoff value to classify the samples into high- or low-expression groups.

### 2.7. Transwell migration assay

NSCLC cells were transfected with DLAT-vector, DLAT sequence, si-DLAT#2, si-DLAT#3 plasmids. The migration abilities of transfected NSCLC cells were assessed using transwell chambers, following the previously described methodology.^[9]^ After 48 hours of incubation, cells in the upper chamber were fixed, stained, and then counted under a microscope.

### 2.8. Statistics analysis

All statistical analyses were performed using R software (version 4.1.2) if not otherwise specified. The data were visualized by ggplot2. Statistical analyses were performed using Statistical Package for the Social Sciences (version 26) in IHC validation section. The chi-square test was used to analyze the correlation of categorical data. Kaplan–Meier survival analysis was used to assess the survival of NSCLC. *P* ≤ .05 was considered statistically significant.

## 3. Results

### 3.1. Differential and prognostic analysis of CRGs

**Figure 1.**
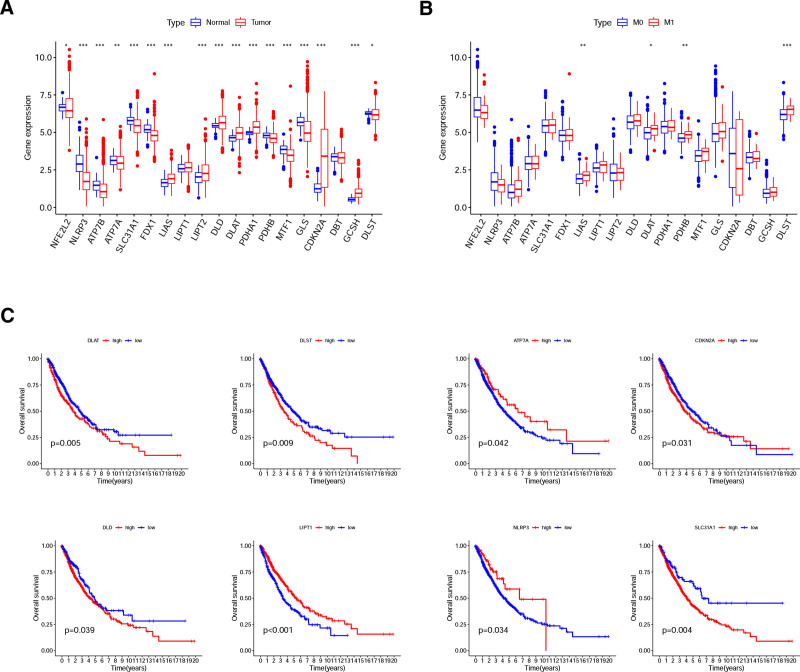
Differential analysis and prognostic analysis of CRGs. (A) Differential analysis of CRGs in normal and NSCLC tissues (blue: normal tissues; red: NSCLC tissues). (B) Differential analysis of CRGs between M0 and M1 (blue: M0; red: M1). (C) CRGs significantly associated with prognosis (blue: low expression; red: high expression). *P < .05; **P < .01; ***P < .001. CRGs = cuproptosis-related genes, NSCLC = non-small cell lung cancer.

Gene expression profiles and clinical information of 99 normal lung tissues and 932 NSCLC tissues were obtained from the TCGA database. Differential analysis was performed based on the expression of 19 CRGs, and 17 differentially expressed CRGs were determined after comparing normal and tumor tissues. Among them, 7 CRGs (LIAS, LIPT2, DLD, DLAT, PDHA1, CDKN2A, and GCSH) were up-regulated in NSCLC tissues, while 10 CRGs (NFE2L2, NLRP3, ATP7B, ATP7A, SLC31A1, ferredoxin 1 [FDX1], PDHB, MTF1, glutaminase [GLS], and DLST) were down-regulated (Fig. 1A). Seven hundred ninety-nine of 932 NSCLC cases had complete metastasis data, and we grouped them into 2 subgroups (M0 for no metastasis and M1 for metastasis). Four CRGs (LIAS, DLAT, PDHB, and DLST) were over-expressed in the M1 subgroup (Fig. 1B). In addition, 8 CRGs (DLAT, DLST, ATP7A, CDKN2A, NLRP3, SLC31A1, DLD, and LIPT1) were significantly associated with prognosis (*P* < .05). Notably, DLAT and DLST were differentially expressed in the M1 subgroup and also remarkably correlated with prognosis (Fig. 1C).

### 3.2. Identification of cuproptosis clusters based on CRGs

**Figure 2.**
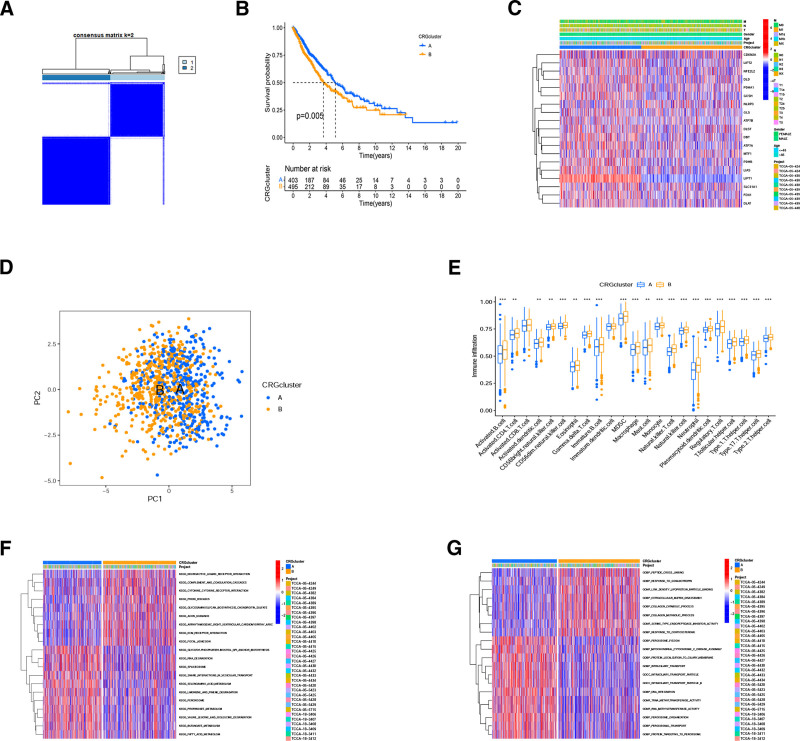
Tumor consensus clustering analysis based on CRGs. (A) NSCLC samples were classified into 2 clusters utilizing the consensus clustering matrix (k = 2). (B) Comparing OS rates via KM survival analysis for the 2 clusters. (C) The heatmap depicting the clinicopathological parameters and CRGs expression of the 2 clusters. (D) PCA plot for NSCLC patients depending on clusters. (E) GO function analysis in the 2 clusters. (F) KEGG pathway analysis in the 2 clusters. CRGs = cuproptosis-related genes, GO = gene ontology, NSCLC = non-small cell lung cancer, OS = overall survival, PCA = principal component analysis.

To further understand the relationship between CRGs and NSCLC, 2 cuproptosis clusters were identified (cluster A and cluster B) (Fig. 2A). The survival of cluster A was significantly better than cluster B (Fig. 2B). Little difference in the clinical parameters between clusters A and B were found, but the expression of CRGs were higher in cluster A (Fig. 2C). It can be speculated that the survival difference between clusters A and B was explained by the differential CRGs expression. This further supported that CRGs played a certain role in NSCLC. Principal component analysis analysis indicated that the 2 cuproptosis clusters could be distinguished, but there was partial overlap (Fig. 2D), considering to be caused by our selection of only 2 principal components. The ssGSEA demonstrated that all immune cells, except CD8+ T cells and immature dendritic cells, had lower infiltration degree in cluster A than in cluster B (Fig. 2E). The heart of the tumor immune response is CD8+ T cells, and this suggested that CRGs may influence the progression and prognosis of NSCLSC by regulating immune cell infiltration apart from CD8+ T cells. However, which and how CRGs regulate the relevant immune infiltrating cells requires further study. GSVA revealed that the 2 subclusters were significantly different on the functional enrichment, which may account for the prognostic difference (Fig. 2F and G). Cluster A was enriched in intraciliary transport, RNA methylation, and metabolism of fatty acids and amino acids. While cluster B was enriched in ligand–receptor interactions and collagen metabolism. Total 185 DEGs were identified in the 2 subclusters. They were mainly enriched on immunological functions, IL-17 signaling, extracellular matrix (ECM)-receptor interactions, and local adhesion pathways (Fig. S1, Supplemental Digital Content, http://links.lww.com/MD/O98). These functions and pathways were mainly focused on immune response and tumor metastasis.

### 3.3. Construction and validation of a prognostic model based on CRGs

**Figure 3.**
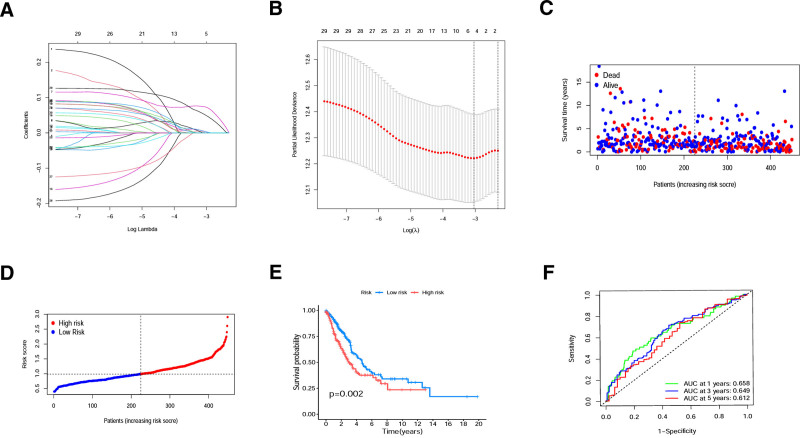
Construction of risk signature in the train cohort. (A) LASSO regression analysis of DEGs. (B) Cross-validation results of Lasso Cox regression analysis. (C) Distributions of OS status, OS, and risk scores. (D) Distribution of patients based on the risk score (low-risk population: on the left side of the dotted line; high-risk population: on the right side of the dotted line). E. Kaplan–Meier curves of patients in the high-risk and low-risk groups. (F) ROC curves demonstrated the predictive efficiency of the risk score. DEG = differentially expressed gene, OS = overall survival, ROC = receiver operating characteristic.

A risk score prognostic model was constructed (Fig. 3A and B) and the equation is as follows: risk score$=\;\left(Exp_{SERPINE1}\times 0.143657491357375\right)$$+\left(Exp_{FAM83A}\times 0.104404411929857\right)$. The risk scores were calculated for each individual according to the formula, and patients were divided into a high- or low-risk group based on the median risk score (Fig. 3C and D). The genes in the prognostic model were both highly expressed in the high-risk group (Fig. S2A–C, Supplemental Digital Content, http://links.lww.com/MD/O98). The Sankey diagram was used to visualize differences in risk scores and in survival status among the subclusters (Fig. S2D, Supplemental Digital Content, http://links.lww.com/MD/O98). The majority of patients in cluster A were in the low-risk group, and most of them were still alive by the date of investigation. This was consistent with the fact that cluster B had a higher risk score than cluster A significantly (Fig. S2E, Supplemental Digital Content, http://links.lww.com/MD/O98).

**Figure 4.**
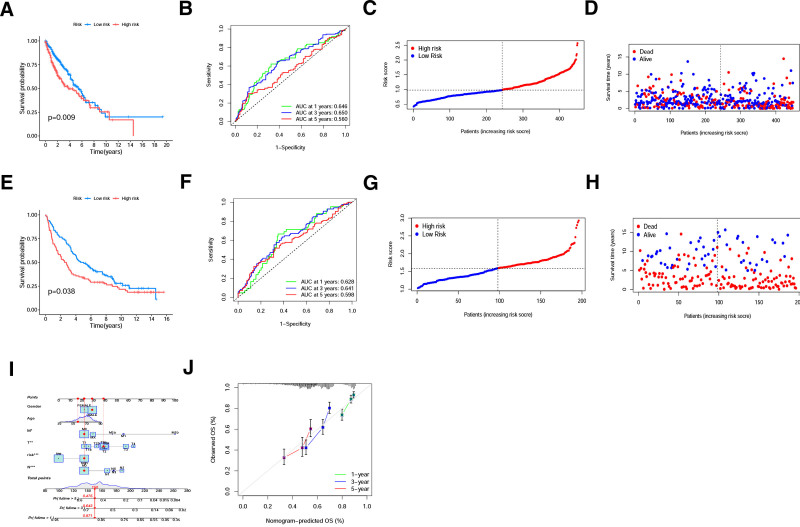
Validation of the risk model. (A) Kaplan–Meier curves between low-risk and high-risk groups in TCGA internal testing set. (B) ROC curves for the prognostic model in TCGA internal testing set. (C) Distribution of patients in internal testing set. (D) Distributions of OS status, OS, and risk scores in TCGA internal testing set. (E) Kaplan–Meier curves between low-risk and high-risk groups in GEO external testing set. (F) ROC curves for the prognostic model in GEO external testing set. (G) Distribution of patients in the GEO external testing set based on risk scores. (H) Distributions of OS status, OS, and risk scores in GEO external testing set. (I) The total score of the patient in the nomogram was 150, which indicated that the survival probability of this patient at 1-, 3-, and 5-years was 87.1%, 64.3%, and 47.5%, respectively. (J) Calibration plots for agreement tests between predicted and actual OS. GEO = gene expression omnibus, OS = overall survival, ROC = receiver operating characteristic, TCGA = The Cancer Genome Atlas.

To validate the capability of the model to predict prognosis, Kaplan–Meier survival analysis and ROC curve analysis were performed in the training set, TCGA internal testing set and GEO external testing set. Patients in TCGA internal testing set and GEO external testing set were also divided into a high- or low-risk group based on the median risk score (Fig. 4C, D, G and H). The survival rates of patients in the low-risk group were significantly higher than those in the high-risk group (Figs. 3E and 4A and E). The ROC curves indicated that the model has a good predictive capability (Figs. 3F and 4B, F). The multivariable Cox regression was visualized by nomogram, which showed that the risk score was an independent prognostic factor for NSCLC (Fig. 4I). The calibration curves showed that the actual curves were close to the predicted curves, which confirmed the encouraging ability of the constructed model (Fig. 4J).

### 3.4. Analysis of tumor microenvironment and drug sensitivity based on high- and low-risk groups

**Figure 5.**
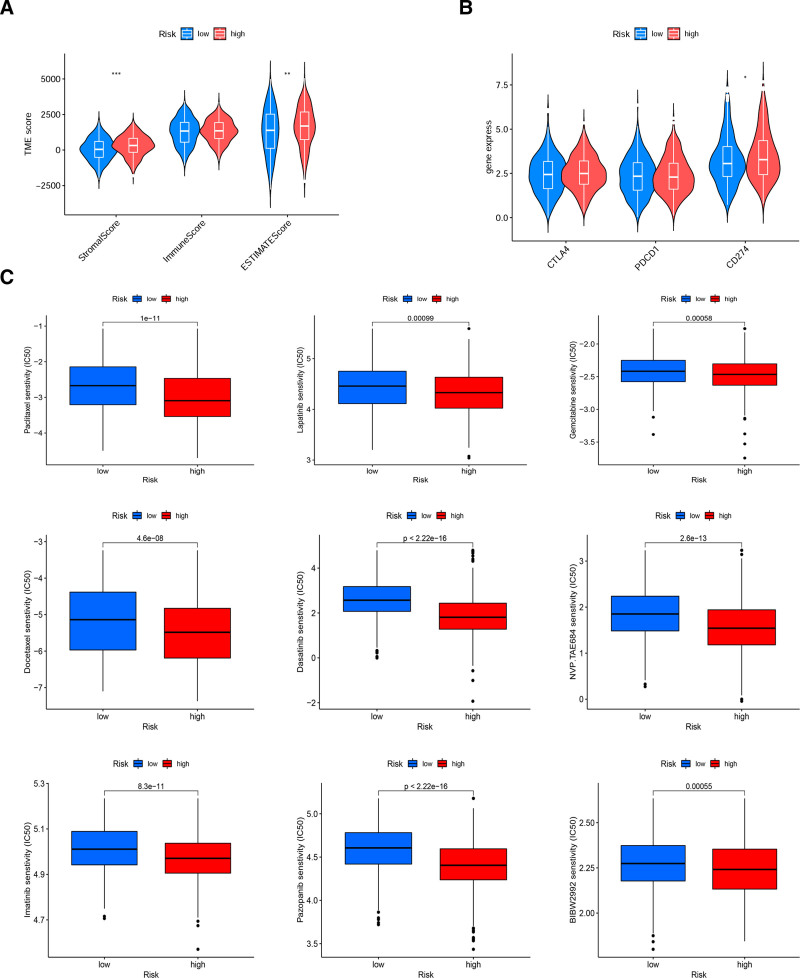
Analysis of tumor microenvironment and drug sensitivity based on high- and low-risk groups. (A) Differential analysis of immune microenvironment scores between high- and low-risk groups. (B) Differential analysis of gene expression of 3 immune checkpoints between high and low risk groups. (C) Some drugs with different sensitivities in high- and low-risk groups. *P < .05; **P < .01; ***P < .001.

The tumor microenvironment (TME) is a complex environment for tumor cells to survive and progress, including immune cells, stromal cells, various signaling molecules and ECM. The stromal and ESTIMATE scores were significantly higher in the high-risk group, while the immune score was not significantly different in the 2 risk groups (Fig. 5A). The tumor purity was negatively correlated with ESTIMATE scores. Therefore, we speculated that the low survival in the high-risk group was a consequence of stromal cells in TME. Previous studies have demonstrated that key components of stroma in TME promote tumor cell growth and metastasis, and also influence immunotherapy and drug sensitivity of tumors. Differential analysis of 3 common immune checkpoint genes showed that the expression of CD274 (known as PD-L1) was significantly higher in the high-risk group, while programmed cell death protein 1 and CTLA4 were not, supporting the potential importance of cuproptosis-related genes in the context of immunotherapy. This illustrated that patients in the high-risk group might achieve greater clinical benefits by using immunotherapy with anti-PD-L1 immunotherapy (Fig. 5B). It also meant that our model may provide information for screening the population for immunotherapy benefits. The half maximal inhibitory concentration (IC50) of common antitumor drugs were calculated in the 2 risk groups (Fig. 5C). The smaller the IC50 was, the greater the sensitivity of the tumor to the drug. Most drugs were more sensitive in the high-risk group, including common chemotherapy drugs such as gemcitabine and paclitaxel-like drugs, as well as common targeted drugs, like tyrosine kinase inhibitors (TKIs). It can be seen that NSCLC in the high-risk group was more sensitive to chemotherapy and targeted therapy.

### 3.5. Combined diagnosis and single-gene batch correlation analysis for DLAT and DLST

**Figure 6.**
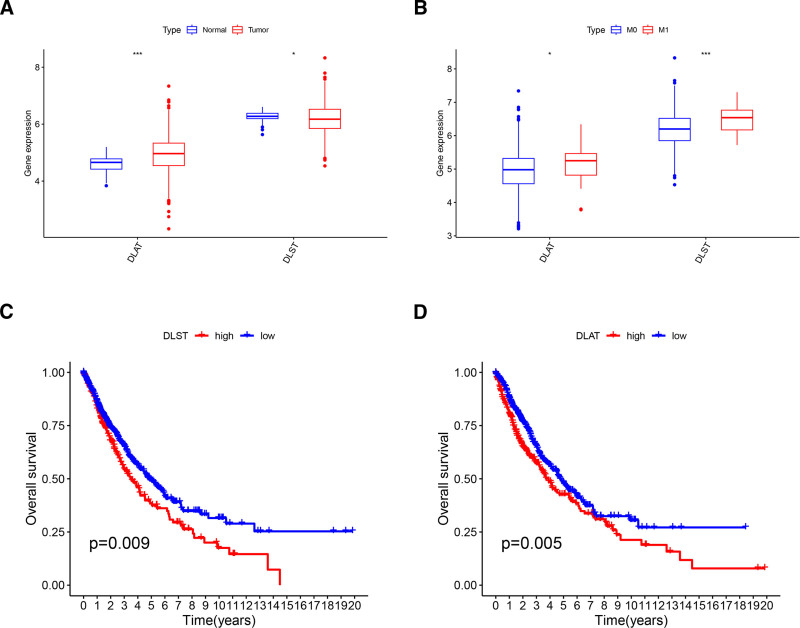
DLAT and DLST were identified hub genes in NSCLC. (A) DLAT and DLST were differentially expressed in NSCLC tissues. (B) DLAT and DLST were differentially expressed in M1 subgroup. (C) High expression of DLAT was significantly associated with poorer prognosis. (D) High expression of DLST was significantly associated with poorer prognosis. *P < .05; **P < .01; ***P < .001. DLAT = dihydrolipoamide S-acetyltransferase, DLST = dihydrolipoamide S-succinyltransferase, NSCLC = non-small cell lung cancer.

Protein lipoylated was a crucial process in cuproptosis, and DLAT and DLST were proven to be essential lipoylated protein genes. In addition, they were differentially expressed both in NSCLC and in the M1 subgroup, and high expression of them was associated with poorer prognosis (Fig. 6). We thus wondered whether the combined diagnosis of DLAT and DLST could predict metastasis in NSCLC. A model was constructed to calculate the probability of metastasis in NSCLC. The AUCs of DLAT and DLST was 0.555 and 0.446 respectively, while the AUC increased to 0.578 after combining the DLAT and DLST. This proved that the combined diagnosis of DLAT and DLST can predict the metastasis in NSCLC to some extent. However, it is unclear how they affect metastasis and prognosis in NSCLC because the functions of them are poorly studied. Genes in the top 1000 correlated with DLAT and DLST were obtained respectively, and gene ontology enrichment analysis was performed on them. DLAT-correlated genes were involved in ATP hydrolysis, GTPase binding, ribonucleoproteins (RNPs) biogenesis, and RNA splicing. DLST-correlated genes were involved in ATP hydrolysis, RNA helicase activity, RNPs biogenesis and RNA splicing (Fig. S3, Supplemental Digital Content, http://links.lww.com/MD/O98). Their involvement in ATP metabolic was consistent with the fact that tumor cells require high energy for metastasis. In addition, they were involved in RNPs biogenesis and RNA splicing, which could also affect tumor cell metastasis.

### 3.6. IHC validation for DLAT and DLST

**Figure 7.**
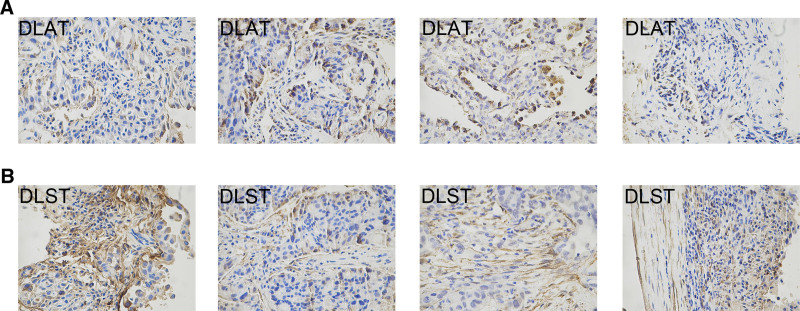
The immunohistochemical staining of DLAT and DLST in NSCLC tissues. (A) DLAT immunohistochemical staining of NSCLC tissues from 4 different patients. (B) DLST immunohistochemical staining of NSCLC tissues from the above 4 patients. DLAT = dihydrolipoamide S-acetyltransferase, DLST = dihydrolipoamide S-succinyltransferase, NSCLC = non-small cell lung cancer.

Immunohistochemical validation was performed on 50 NSCLC samples, 4 cases are shown in Figure 7. The samples were divided into high- or low-expression group based on the median H-Score of DLAT and DLST. The clinicopathological characteristics and their correlation with the expression of DLAT and DLST are shown in Table 1. DLAT expression was significantly correlated with the M stage, but it was not correlated with gender, age, T and N stages, pathology, and brain metastasis status. DLST expression was significantly correlated with T, N, and M stages, pathology, and brain metastasis status, but not age and gender. DLAT and DLST expression were both significantly associated with metastasis, and univariate and multifactorial logistic regression analyses showed that gender, expression of DLAT and DLST were all independent predictive factors for metastasis (Table 2). Males in gender, high DLAT-expressing, and low DLST-expressing of NSCLC were more likely to have metastasis. This aligns precisely with our bioinformatics analysis results, indicating a significantly increased expression of DLAT within the M1 subgroup. However, the IHC suggested that DLST expression was lower in the M1 subgroup, which probably resulted from limitations in sample source. That is, DLAT and DLST expression can predict metastasis in NSCLC.

**Table 1:** Clinicopathological features of 50 NSCLC patients.

Characteristics	Number	DLAT		*P*-value	DLST		*P*-value
		Low	High		Low	High	
Gender							
Male	33	18	15	.551	16	17	1
Female	17	7	10		9	8	
Age/years							
<60 years	18	8	10	.769	7	11	.377
≥60 years	32	17	15		18	14	
Pathology							
Squamous carcinoma	5	3	2	1	0	5	.05
Adenocarcinoma	45	22	23		25	20	
T							
T1–2	37	18	19	1	23	14	.008
T3–4	12	6	6		2	10	
Tx	1	1	0		0	1	
N							
N0	10	3	7	.306	9	1	.01
N1–3	36	18	18		14	22	
Nx	4	4	0		2	2	
M							
M0	25	8	17	.023	18	7	.004
M1	25	17	8		7	18	
Brain metastasis							
Yes	5	3	2	1	0	5	.05
No	45	22	23		25	20	
PD-L1							
<1%	20	7	13	.039	11	9	.831
1–49%	16	7	9		8	8	
≥50%	14	11	3		6	8	

NSCLC = non-small cell lung cancer.

**Table 2:** Univariate and multifactorial logistic analysis of metastasis.

Items	Univariate analysis			Multivariate analysis		
	HR	95% CI	*P*-value	HR	95% CI	*P*-value
Gender	3.692	1.052–12.957	.041	4.802	1.015–22.718	.048
DLAT	4.516	1.376–14.820	.013	4.037	1.022–15.954	.047
DLST	0.151	0.044–0.520	.003	0.135	0.032–0.559	.006

In addition, the expression of DLST showed a correlation with brain metastasis status (*P* = .05), so DLST can help clinicians identify high-risk brain metastasis patients and thus prevent brain metastasis in advance. The middle progression-free survival (PFS) was 14 and 25 months (*P* = .134) for the DLAT high and low-expression groups, and 22 and 24 months (*P* = .226) for the DLST high and low-expression groups. This means that both DLAT and DLST were not significantly different in survival. Among the 24 NSCLC patients with immunotherapy, the middle PFS for DLAT high and low expression groups were 7 and 24 months (*P* = .018), and the middle PFS for DLST high and low expression groups were 15 and 12 months (*P* = .552). This suggested that DLAT might predict NSCLC immunotherapy efficacy, consistent with a fact that DLAT expression was significantly correlated with PD-L1 expression (*P* = .039).

### 3.7. DLAT expression affects cell migration in NSCLC cells

**Figure 8.**
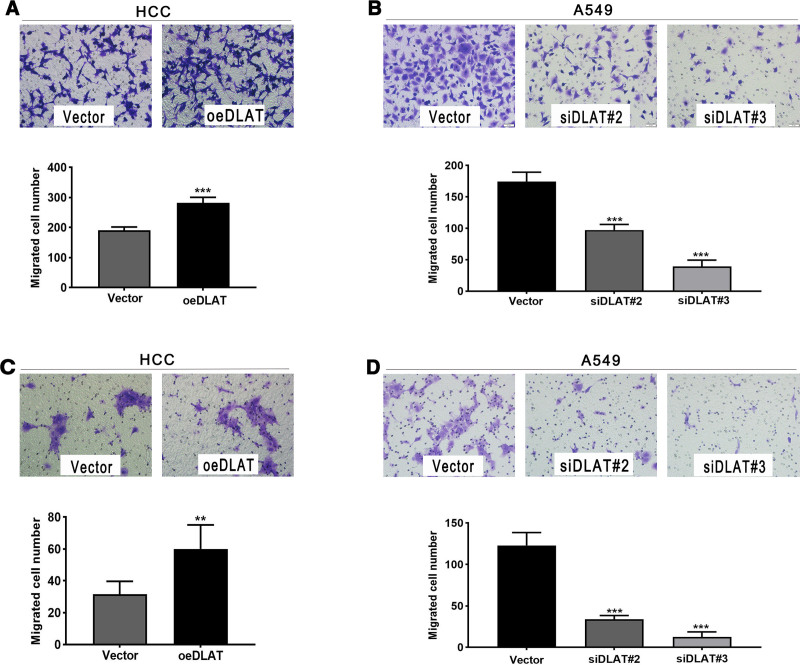
DLAT modulates migration in NSCLC cells. (A) Migratory ability of A549 cells after DLAT overexpression. (B) Migratory ability of A549 cells after DLAT knockdown. (C) Migratory ability of HCC827 cells after DLAT overexpression. (D) Migratory ability of HCC827 cells after DLAT knockdown. DLAT = dihydrolipoamide S-acetyltransferase, DLST = dihydrolipoamide S-succinyltransferase, NSCLC = non-small cell lung cancer.

From the transwell assay, the number of migration cells were significantly increased in A549 and HCC827 cells transfected with oeDLAT compared to cells transfected with the vector control (Fig. 8A and C). Subsequently, we knocked down DLAT in A549 and HCC827 cells, and assessed the migrated cell number in these cell lines. As depicted in Figure 8B and D, the knockdown of DLAT led to a reduction in cell migration in both A549 and HCC827 cells compared to the vector control-transfected cells. Notably, knockout of DLAT significantly reduced the proliferation capabilities of A549 and HCC827 cells (*P* < .01). Similarly, overexpression of the gene led to increase in cell proliferation rate (*P* < .001).

### 3.8. An illustrative case of DLAT for predicting immunotherapy efficacy in NSCLC

**Figure 9.**
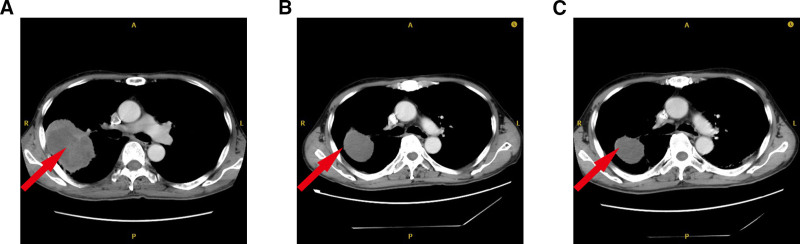
An illustrative case of a 61-year-old NSCLC man with anti-PD-L1 immunotherapy on March 2, 2021. (A) Chest enhancement CT before treatment on January 26, 2021, the tumor size was 8.31 × 6.17 cm. (B) Chest enhancement CT after 4 months of immunotherapy on May 26, 2021, tumor size was 5.61 × 4.53 cm. (C) Chest enhancement CT after 9 months of immunotherapy on October 20, 2021, tumor size was 4.70 × 3.93 cm. CT = computed tomography, NSCLC = non-small cell lung cancer, PD-L1 = programmed death-ligand 1.

To demonstrate visually the therapeutic response of NSCLC lesions to anti-PD-L1-immunotherapy, we examined an illustrative case who showed a low DLAT expression. A 61-year-old NSCLC patient received anti-PD-L1-immunotherapy. Computed tomography showed the lung tumor size of 8.31 × 6.17 cm before the patient received immunotherapy. 9 months after treatment, computed tomography revealed the tumor size decreased to 4.70 × 3.93 cm (Fig. 9). The patient’s condition was assessed for PR according to the iRECIST evaluation criteria. This further demonstrates that low DLAT expression predicts good immunotherapy efficacy.

## 4. Discussion

Previous studies have revealed that Cu is closely involved in cell proliferation, tumor metastasis, angiogenesis and the remodeling of tumor microenvironments via various molecular mechanisms.^[10,11]^ Moreover, Cu can regulate the expression of PD-L1 in tumor cells, thus affecting anti-PD-L1 antitumor therapy.^[12]^ The above studies all illustrate the importance of Cu in tumor cells. Recently, it was found that excess intracellular copper can trigger a new form of cell death by targeting lipoylated proteins in the TCA cycle. This means that Cu combines with lipoylated proteins, causing aggregation of lipoylated protein and loss of iron-sulfur cluster protein. It then leads to proteotoxic stress and ultimately cell death.^[3]^ This particular type of cell death is known as cuproptosis and is receiving increasing attention. However, the prognostic value of CRGs in NSCLC is unknown. DLAT and DLST are essential lipoylated protein genes in the TCA cycle. As shown in the part of our bioinformatics analysis, DLAT and DLST played an essential role both in the metastasis and prognosis of NSCLC. However, the result is only based on public databases and still need to be supported by experiments and clinical data. Therefore, our study not only explored the prognostic value of the CRGs-related prognostic model but innovatively analyzed the value of DLAT and DLST in metastasis for NSCLC.

In our study, we identified 2 cuproptosis clusters based on CRGs, where patients in cluster A had a significantly better prognosis than cluster B. CRGs in NSCLC are upregulated in the majority of cluster A, with LIPT1 most notably. It has been reported that LIPT1 is upregulated in melanoma and is an independent favorable prognostic indicator.^[13]^ Therefore, LIPT1 may play a similar role in inhibiting tumor progression in NSCLC. In addition, 185 DEGs were identified in these 2 clusters. The above results initially confirm the potential value of CRGs in NSCLC.

Our risk score prognostic model ultimately identified 2 genes, Serpin Family E Member 1 (SERPINE1) and FAM83A. SERPINE1, an inhibitor of plasminogen activator (PA), was expected to protect against tumor proliferation by inhibiting PA activity. However, several studies have confirmed that SERPINE1 was significantly upregulated in a variety of malignancies, including glioblastoma, esophageal squamous carcinoma, breast cancer, gastric cancer, bladder cancer, and oral squamous carcinoma. It is also an important marker of poor prognosis in various malignancies.^[14–20]^ Therefore, Miguel et al^[21]^ concluded that SERPINE1 was responsible for the poor prognosis by multiple signaling pathways which were independent of the PA system. FAM83A, a family member A gene with sequence similarity 83, was identified as a tumor-promoting gene that is upregulated in several malignancies and was significantly associated with poor prognosis in tumors.^[22–26]^ In addition, overexpression of FAM83A may affect PD-L1 expression and resistance to EGFR-TKI drugs, which may affect the efficacy of immunotherapy and targeted therapy.^[27,28]^ It was evident that the 2 genes included in the risk model were closely related to the progression and prognosis of tumors. For this reason, it is well-supported to use them to construct the prognostic models.

NSCLC is a highly heterogeneous tumor with relatively complex treatment and highly different survival rates. The treatment and prognosis of NSCLC differ markedly by individual, even if identical in the stage. In clinical practice, molecular pathology test results of NSCLC are used to predict the development of NSCLC and help clinicians to individualize treatment, but they are not completely accurate. We classified NSCLC into high- and low-risk groups based on the risk prognostic score model. The survival of the low-risk group is remarkably better than the high-risk group. Moreover, risk score was an independent risk factor for NSCLC prognosis. All of these supported the potential clinical value of the risk score. In tumor tissues, the purity of tumor cells was lower in the high-risk group for NSCLC, while the immune infiltration did not differ significantly compared with the low-risk group. It suggested that the poorer prognosis in the high-risk group may be explained by the stromal component in the tumor tissues. Some components of the stroma, such as cytokines produced by cancer-associated fibroblasts and tumor-associated macrophages, promote infiltration and metastasis of tumor cells.^[29]^ In general, an increase in stromal components, such as ECM, may increase drug resistance in tumor cells. However, our study showed that the high-risk group was more sensitive to some common antitumor drugs. It indicates that CRGs may affect the genetics or epigenetics of tumor cells rather than TME, resulting in higher drug sensitivity in the high-risk group. Although this study did not directly explore the relationship between the cuproptosis-related gene signature and other known prognostic factors in NSCLC, we found that the high-risk group in the signature was associated with high PD-L1 expression and high sensitivity to TKIs. This finding suggests that cuproptosis-related genes may indirectly affect patient prognosis through their impact on other prognostic factors. In summary, the model we constructed could be a valuable tool for risk classification of NSCLC. It has a good ability to predict the prognosis of NSCLC and to guide clinical individualized treatment. Future research will need to further explore the specific relationships between cuproptosis-related genes and other biomarkers such as EGFR, Kirsten Rat Sarcoma Viral Oncogene, and anaplastic lymphoma kinase to determine their combined roles in NSCLC prognosis and treatment. Additionally, exploring how these genes interact in different treatment response backgrounds could reveal their potential as markers for monitoring treatment efficacy or as therapeutic targets.

Currently, most of the metastases in NSCLC are clinically diagnosed through imaging. However, imaging is difficult to confirm early metastases and there is also a certain rate of false positives and false negatives. This leads to the clinicians’ failure to detect the sign of metastasis in time, so there is a demand for higher diagnostic efficacy of metastasis in clinical practice. Immunotherapy based on immune checkpoint inhibitors led to a leap forward in the treatment of NSCLC, increasing the 5-year survival rate of NSCLC to 16% for the first time, but only some of the patients benefit from immunotherapy. PD-L1 testing has been included in the treatment guideline for NSCLC for screening potential beneficiaries of immunotherapy. However, PD-L1 testing is still an effective but imperfect tool and not an absolute marker for immunotherapy due to various limitations.^[30]^

In this study, we used bioinformatics to discover that DLAT and DLST play an important role in metastasis in NSCLC. However, there are no studies on their role in tumor metastasis. Single-gene bulk correlation analysis indicated that the functions of them have a large partial overlap and were mainly focused on the processes of RNA splicing and processing and synthesis of RNPs. It has been reported that dysregulation of the RNA splicing was associated with increased invasion, angiogenesis, metastasis, and drug resistance of cancer cells.^[31]^ In addition, RNPs may also influence tumor progression through diverse mechanisms. Interestingly, the spliceosome is one of the members of RNPs. Moreover, growing evidence strongly identifies an essential link between the epithelial–mesenchymal transition program and ribonucleoprotein (RNP) biogenesis,^[32,33]^ which leads to tumor cell migration, invasion, and eventual metastasis. Previous studies shows that high expression of DLAT is associated with metastasis in liver and colon cancer, predicting a poor prognosis.^[34,35]^ Depletion and inhibition of DLST decrease invasion and metastasis of triple-negative breast cancer cells.^[36]^ In conclusion, these studies provide a theoretical basis for our study that explores the value of DLAT and DLST in NSCLC metastasis.

The results of IHC analysis were generally consistent with the results of the bioinformatic analysis. DLAT and DLST expressions were significantly correlated with metastasis and have the potential to be markers for predicting metastasis in NSCLC. Moreover, both DLAT and DLST expression were independent predictive factors for metastasis in NSCLC. In the transwell migration assay, we observed that the overexpression of DLAT significantly increased the migratory capacity of cells in vitro, while the knockdown of DLAT effectively inhibited cell migration. These results contribute to a better understanding of the molecular mechanisms underlying the metastatic process in NSCLC and may have implications for the development of targeted therapies. However, these results are limited to in vitro experiments. To further explore the molecular mechanisms of DLAT, additional in vivo experiments and molecular pathway studies are needed.

Our study has thoroughly analyzed the functions of DLAT in NSCLC, finding that the knockout or overexpression of the gene significantly affects cell proliferation and invasion capabilities. Although our study did not directly analyze FDX1 and GLS-other cuproptosis hub genes, the latest research indicates that their roles in cuproptosis are equally important. Research indicates that FDX1 plays a crucial role in cellular redox reactions, impacting NSCLC cell survival and death. Lower expression levels of FDX1 have been associated with poor prognosis in patients.^[37]^ Additionally, GLS, a key enzyme in glutamate metabolism, is often upregulated in NSCLC. The increased activity of GLS meets the energy and biosynthetic precursor demands of rapidly proliferating tumor cells. Furthermore, inhibition of GLS has shown potential in suppressing tumor growth, highlighting its therapeutic potential.^[38]^ Considering the role of cuproptosis in tumor biology, future research should further explore the functions of FDX1, GLS, and other related genes, assessing their potential as therapeutic targets. Additionally, exploring how to optimize treatment strategies for NSCLC patients by modulating Cu concentrations or targeting specific cuproptosis pathways will be a valuable research direction.

NSCLC patients have a high rate of brain metastasis, which response poorly to conventional drug therapy due to the presence of the blood–brain barrier. Fortunately, we found that DLST expression has the potential to predict brain metastasis, so clinicians may expect to selectively take measures to prevent brain metastasis based on DLST expression, thus making treatment more individualized. This may enable more patients to benefit from brain radiotherapy. Furthermore, although our study demonstrated that DLAT and DLST were strongly associated with metastasis, the IHC results showed that none of their associations were reflected in the prognosis. In our study, DLAT expression significantly correlated with PD-L1, and low DLAT expression indicated better immunotherapeutic efficacy, while DLST did not. It has been confirmed that CRGs could predict immunotherapy efficacy for breast cancer.^[39]^ It may provide preliminary evidence for the potential of DLAT in predicting the efficacy of anti-PD-L1 immunotherapy for NSCLC. Therefore, we need more in-depth studies to explore the value of DLAT and DLST in tumor cells and to determine their accuracy and reliability as potential markers.

However, a major limitation of this study is the relatively small sample size. While these samples provided certain insights, the limited sample size may affect the statistical power and the broader applicability of our findings. Therefore, the conclusions of this study need to be validated in future larger-scale research. Although this study’s sample exhibits bias, it still provides important insights into the role of cuproptosis in NSCLC, and these findings lay the foundation for future research on a broader and more diverse sample base. Future research should include more squamous cell carcinoma patient samples to validate the universality of the current findings and explore differences between subtypes.

In conclusion, the prognostic model constructed based on CRGs has a good predictive ability for the prognosis of NSCLC, and may provide a reference for the clinical interventions. DLAT may play a role in predicting immunotherapy efficacy. In addition, DLAT and DLST are expected to serve as markers of metastasis in NSCLC.

## Acknowledgments

The authors would like to thank the reviewers for their helpful comments on this article, as well as the TCGA and GEO databases for kindly providing the data.

## Author contributions

**Investigation:** Yizhi Ge.

**Methodology:** Tingting Wang, Wei Chen.

**Visualization:** Yizhi Ge.

**Writing – original draft:** Huiying Ma, Yuhong Li.

**Writing – review & editing:** Wei Chen, Yizhi Ge.

## Supplementary Material


