# Supplementary figures and images for: Construction of a prognostic model based on cuproptosis-related genes and exploration of the value of DLAT and DLST in the metastasis for non-small cell lung cancer
Source: Medicine (Baltimore). 2024 Dec 6;103(49):e40727. doi: 10.1097/MD.0000000000040727 (PMC11631004; doi:10.1097/MD.0000000000040727)

Figure S1


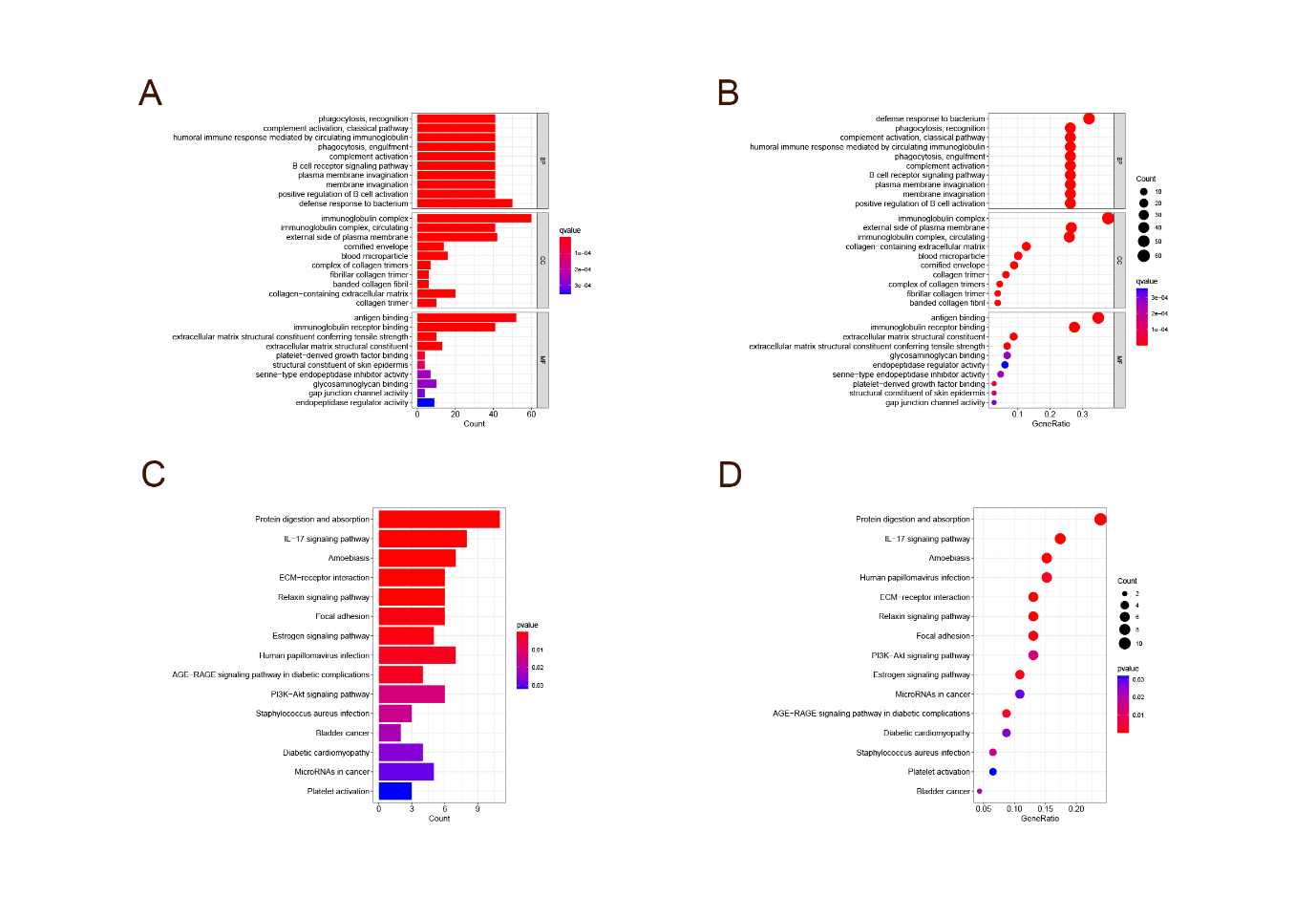


Figure S2


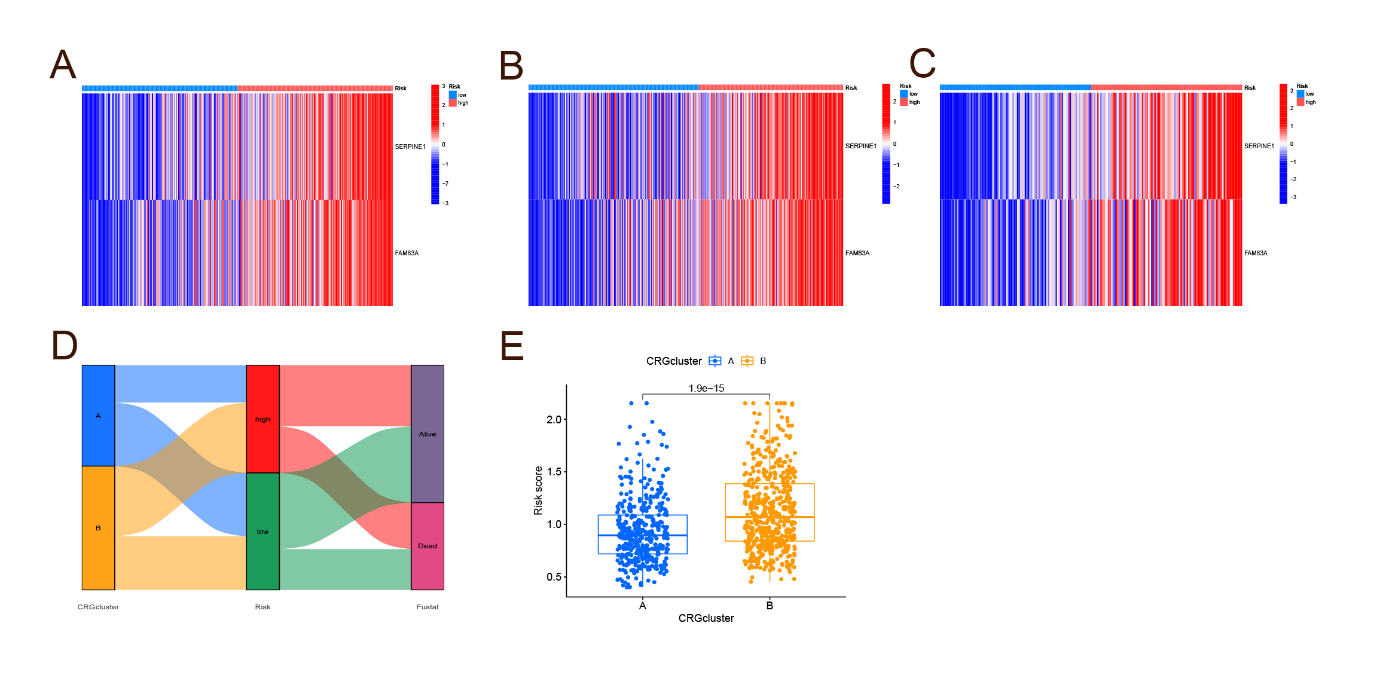


Figure S3


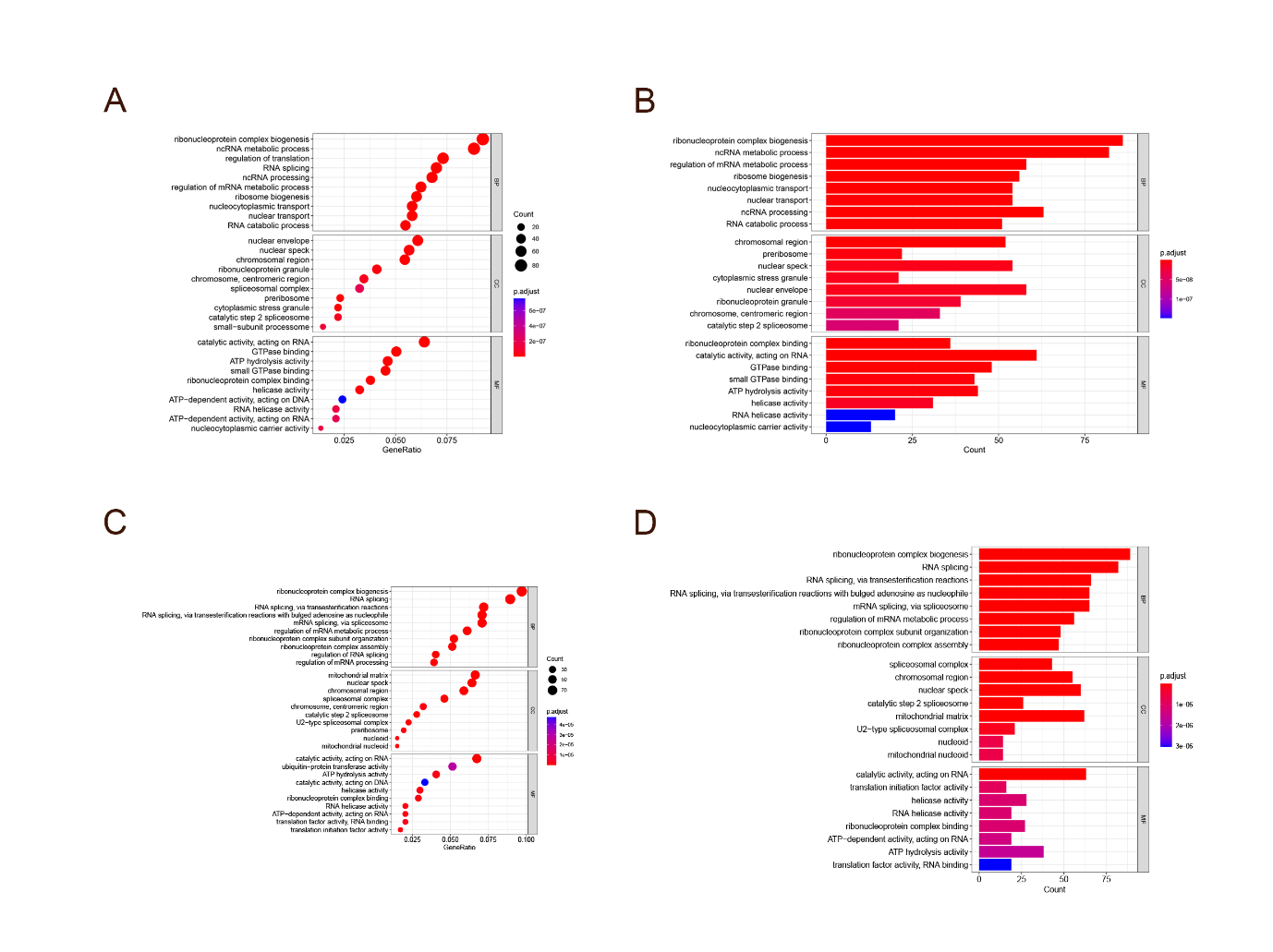

Supplement: Supplementary file 1 [file medi-103-e40727-s001.docx]
